# Signal Enhancement of Cadmium in Lettuce Using Laser-Induced Breakdown Spectroscopy Combined with Pyrolysis Process

**DOI:** 10.3390/molecules24132517

**Published:** 2019-07-09

**Authors:** Zhenghui Chen, Tingting Shen, Jingdong Yao, Wei Wang, Fei Liu, Xiaolong Li, Yong He

**Affiliations:** 1 College of Biosystems Engineering and Food Science, Zhejiang University, 866 Yuhangtang Road, Hangzhou 310058, China; 2 Key Laboratory of Spectroscopy Sensing, Ministry of Agriculture and Rural Affairs, Hangzhou 310058, China

**Keywords:** lettuce, cadmium, laser-induced breakdown spectroscopy, pyrolysis, partial least squares regression, support vector regression

## Abstract

Fast detection of heavy metals in lettuce is significant for food market regulation and the control of heavy metal pollution. Advanced methods like laser-induced breakdown spectroscopy (LIBS) technology have been tried to determine the cadmium (Cd) content. To retard the negative effect of complex matrix composition from samples and improve quantitative performance of LIBS technology, the pyrolysis process combined with LIBS was adopted to determine the cadmium (Cd) content of lettuce. Adaptive iteratively reweighted penalized least squares (airPLS) was used to preprocess the LIBS spectra and solve the baseline drift. For multivariate linear regression based on the three selected Cd emission lines correlation coefficient in the prediction set *R_p_*^2^ increased from 0.9154 to 0.9969, and the limit of detection (LOD) decreased from 9.1 mg/kg to 0.9 mg/kg after the pyrolysis process. The partial least squares (PLS) regression and support vector regression (SVR) were applied to construct calibration models based on full spectra. In addition, the least absolute shrinkage and selection operator (LASSO) was implemented to choose limited lines to predict the Cd content. The PLS model with the pyrolysis process obtained the best results with *R_p_*^2^ = 0.9973 and LOD = 0.8 mg/kg. The results indicated that the pyrolysis method could enhance the spectral signal of cadmium and thus significantly improve the analysis results for all the models. It is shown in this experiment that proper sample preprocessing could effectively amplify the Cd signal in LIBS and make LIBS measurement an efficient method to assess Cd contamination in the vegetable industry.

## 1. Introduction

With the exponential population growth and rapid development of industrialization, the problem of environmental pollution is getting worse, especially the toxic heavy metal contamination such as mercury, lead, cadmium, zinc, copper, nickel, and chromium [1]. As one of the most toxic heavy metals, cadmium (Cd) is attracting worldwide attention because it is non-biodegradable and widespread in water, soil and the other environment [2]. The accumulation of heavy metal Cd in the environment finally cause adverse effects on human health through the food chain. Heavy metal Cd can cause undesirable effects and severe problems for the human body even at very low concentrations, including cancer, mutation, renal failure and chronic anemia [3,4,5].

Lettuce is a common vegetable which represents the most frequently and worldwide consumed green leaf vegetable [6]. However, it has been found that lettuce which grows in the sludge-treated soils will have a high accumulation of cadmium in its leaves [7], this characteristic indicates that it can be used as the indicator crop to measure and estimate the level of cadmium contamination in the soil. Therefore, lettuce is one of the most important sources of trace metal intake through the diet [8]. Since high concentrations of cadmium in lettuce are associated with adverse health effects [9], the fast and accurate detection of the lettuce cadmium pollution is highly needed to ensure the food safety, supervise the growth environment of crops and reduce the risk of Cd poisoning.

Conventional methods for detecting heavy metal Cd includes the atomic absorption spectrometry (AAS), inductively coupled plasma with mass spectrometry (ICP-MS) and inductively coupled plasma optical emission spectroscopy (ICP-OES) [10]. Although these traditional methods are sensitive and accurate, they need complex sample preparation steps and produce large amounts of toxic waste, so they are time-consuming and have expensive costs. These shortcomings make it hard to achieve the rapid detection of heavy metals and real-time monitoring, which may not satisfy the demand of rapid screening analysis at the production site [11].

LIBS is a promising optical technique that is highly applicable to food analysis due to its multi-elemental recognition ability, real time analysis, less destructive and least requirement for sample preparation [12]. LIBS has been widely used in food quality detection and control, as well as mineral composition analysis and bacteria contamination [13]. LIBS has been investigated by researchers as a rapid, micro-destructive food analysis tool and used in many different food detection areas, such as the Ca content in comminuted poultry meat [14], Cu determination in orange peel [15], Mg determination in wheat flour [16], and mineral composition in milk [17]. As for the Cd analysis in lettuce, we have several results in the previous study, which indicate that LIBS coupled with chemometrics could be used as a fast, efficient and low-cost method to assess Cd contamination in the vegetable industry [18]. 

However, the performance of the model still needs to be improved, as challenges remain in terms of sample preparation, matrix effects, spectral pre-processing and model calibration. The accuracy and precision of the quantitative analysis can be affected by atmospheric conditions and the properties of samples [19]. The plant tissue is a complex substance, and the changes in chemical composition and physical properties of plant tissue will influence the signal acquired by LIBS. The effect of the complex matrix of samples on the LIBS signal is generally referred as the matrix effect [20]. The physical properties of the sample influence the processes of sample preparation like grinding, screening and tablet pressing, while the chemical properties such as various chemical elements may have an effect on both spectral and non-spectral interferences [21]. In our experiment, the non-spectral interferences of matrix effects are the main problem and need to be eliminated. The pyrolysis of the lettuce sample may be a good way to improve the properties of the samples, reduce the matrix effect and enhance the heavy metal Cd signal in the LIBS spectra, which will be conducive to the improvement of the previous Cd content concentration prediction model. 

In this study, we aim to improve the calibration model for the Cd quantitative analysis in lettuce based on the LIBS technology by attenuating matrix effects. The specific objectives of this study were (1) to accomplish the pyrolysis of the samples and use adaptive iteratively reweighted penalized least squares (airPLS) methods to preprocess raw LIBS spectra; (2) to obtain the partial least squares (PLS) regression model and support the vector regression (SVR) model using multi-variables; (3) to compare the performance of the model and evaluate the effect of the preprocess of raw data and the pyrolysis of the sample.

## 2. Materials and Methods

### 2.1. Sample Preparation

In order to obtain experimental materials for this experiment, lettuce (*Lactuca sativa L. var. longifolia*) leaves were cultivated with different degrees of cadmium stress. The specific preparation process refers to the previous paper [18]. Romaine lettuce seeds were from the Qingxian Chunfeng vegetable variety breeding farm (Cangzhou, Hebei, China). After being sterilized with 1% NaClO solution for 25 min and rinsed with sterile distilled water, the seeds were then germinated on a sterile Murashige and Skoog culture medium at 35 °C and 65% relative humidity for five days until the roots grew to approximately 3 cm. Then, the seedlings were transplanted into 10 L full strength Yamazaki’s nutrient solution [22], which would be renewed every three days. Growth conditions were adjusted to 27/22 °C (16:8 h light-dark cycle), 65% relative humidity and a light intensity of 200 ${\mu \mathrm{mol}\;m}^{-2}{\;s}^{-1}$ [23]. After nine days, plants with similar growing states were treated with four different cadmium solutions (10, 30, 60, and 100 μM cadmium prepared by $CdCl_{2}$ solution). The concentration of Cd is referenced [24,25,26]. After 30 days’ treatment, lettuce leaves were dried at 60 °C for 5 h in an oven, and ground to powder separately. One hundred and fifty milligrams of single lettuce powders were pressed into a square pellet by a tablet pressing machine (FY-24, SCJS, Tianjin, China) with a pressure of 600 MPa for 30 s. Finally, five samples were prepared for each four different Cd concentration groups, and 20 pellets are used for the LIBS analysis.

### 2.2. LIBS Measurements

This experiment used a self-assembled LIBS device [27]. A laser pulse with maximum energy of 200 mJ and 8 ns pulse width were generated at 532 nm by Q-switched Nd: YAG pulse laser (Vlite 200, Beamtech, Beijing, China). A self-made optical system was used to deliver the laser beam to a plano-convex lens (*f* = 100 mm) which focused the beam on the pellet surface. The laser ablated the sample mass and generated plasma which diffused outward to emit electromagnetic waves. Then the waves were collected by an optical fiber and received by the spectrometer (SR-500i-A-R, Andor Technology, Belfast, UK) combined with an intensified charge coupled device (ICCD) camera (DH334T-18F-03, Andor Technology, Belfast, UK). The spectra between 211.92–232.90 nm with 0.02 nm resolution were collected. To control the delay time between the ICCD camera and laser Q-switch, a delay generator (DG645, Stanford Research Systems, Sunnyvale, CA, USA) was used. We set the optimal experimental parameters before the experiment, with a laser energy of 60 mJ, delay time of 1.5 μs and gate width of 10 μs. Lettuce pellets were placed by an automatic x-y-z positioning system to maintain the laser ablation path with 4 × 4 array craters and each crater had five times accumulation of laser pulses. The distance between locations in the sample is 2 mm. An average of the 80 spectra (4 × 4 × 5) was recorded as the spectrum for each sample to reduce fluctuation. The laser pulse frequency range in this study was 1-10 hz, and 1 hz was used in this study. All samples were measured on the same day. The samples were divided into four groups due to different Cd concentrations, and the samples in each group were marked in random order. The LIBS data was acquired one by one from Group 1 to Group 4.

### 2.3. Pyrolysis Process

After LIBS measurements, the tablets were reground to powder. In order to remove residual water and decompose small organic matters in the sample, the powder would be treated by pyrolysis. The samples were put into the muffle furnace (SX-1000, CEF, Tianjin, China) and were heated at a rate of 5 °C/min. The heating was stopped immediately when the temperature reached 250 °C, which only took 45 min. The weight of all the samples before and after treatment was recorded. The samples were stored in the incubator, where the temperature was controlled at 25 °C and the humidity below 5%. Next day, the samples were pressed into square pellets and were measured by LIBS.

The reference Cd contents of lettuce samples before and after pyrolysis were determined with a flame atomic absorption spectrophotometer (AAS) (AA800, PerkinElmer, Waltham, MA, USA). Sample pretreatment methods before AAS can be found in our previous study [18]. The digested solution obtained after microwave digestion was placed in the AAS inlet for Cd content detection. This step was repeated three times and the AAS data was the average of three injection measurements. The standard material GBW10020 (Beijing, China) and GBW10023 (Beijing, China) were used as a control group to guarantee the analysis quality of AAS. The Cd content of lettuce leaves before pyrolysis and after pyrolysis is shown in Table 1. 

### 2.4. Data Analysis

In this experiment, we tried to use the adaptive iteratively reweighted penalized least squares (airPLS) to correct the baseline drift; PLS regression and SVR were also used for Cd content prediction based on the LIBS spectra data. 

In the multivariate analysis, the baseline drift always blurs signals and thus deteriorates analytical results. A novel algorithm namedairPLS was used by previous studies [28,29] to solve this problem. This method works by iteratively changing weights of sum squares errors (SSE) between the fitted baseline and original signals, and the weights of the SSE are obtained adaptively using the difference between the previously fitted baseline and the original signals.

In the LIBS spectroscopy, different transition processes can produce light radiation of different wavelengths; the wavelength of light radiation produced by different elements may be similar, which would cause overlap between characteristic peaks and lead to the deviation of prediction. The partial least squares (PLS) was first introduced by the Swedish statistician Herman [30] and is a widely used multivariate analysis method. Particularly, PLS regression is still effective when the variables are highly linearly correlated just like our spectroscopy [31].

Support vector regression (SVR) is one of the regression versions of SVM. SVR projects data to a high-dimensional feature space through a nonlinear mapping and perform linear regression in this space [32]. A loss function of the modified distance is introduced by SVR based on the classification model and the cost function ignores the support vectors which are training data close to the model prediction. In this experiment, the radial basis function (RBF) was utilized as the kernel function. Two parameters of SVR need to be adjusted. One is the regularization parameter (*gam*), which determines the tradeoff cost between minimizing the training error and minimizing model complexity. The other is the kernel function parameter (*sig2*), which equals to σ^2^ and sigma defines the non-linear mapping from the input space to some high-dimensional feature space [33]. The two parameters were optimized by a grid-search procedure. The parameters with the minimal value of RMSE were chosen and then adopted to build the model.

The least absolute shrinkage and selection operator (LASSO) is a data dimension reduction method that is applicable to linear and nonlinear cases, which was first introduced by Robert [34]. LASSO select the variables of the sample data based on the penalty method, the original small coefficients are directly compressed to zero and discarded as non-significant variables.

In order to compare with the above algorithms, three Cd emission lines were selected and the multivariate linear regression was performed to predict the Cd content. Data analysis was executed by MATLAB R2016a (The MathWorks, Inc., Natick, MA, USA) [35]. 

### 2.5. Performance Evaluation

The effect of the pyrolysis process was assessed by the relative standard deviation (RSD), signal-to-noise ratio (SNR) and signal-to-background ratio (SBR). RSD is the ratio of the standard deviation to the mean. SNR is defined as the ratio of signal power to the noise power and SBR is the ratio of signal power to the background power. By these standards, the enhancement of pyrolysis to the LIBS signal could be analyzed.

To measure the performance of the above quantitative models for the Cd content detection, the root mean square error of cross validation (RMSECV) and root mean square error of prediction (RMSEP) were used. The RMSE evaluates the deviation between the predicted and the referred content. The limit of detection (LOD) was used to evaluate the sensitivity of PLS models. The calculation method of LOD can refer to our previous research [18]. The correlation coefficient (*R*^2^) was also calculated which stands for the correlation between the element content of the models predicted and the reference Cd content. *R_c_*^2^ and *R_p_*^2^ are correlation coefficients of the calibration set and prediction set respectively, showing the accuracy of calibration and prediction models.

## 3. Results and Discussion

### 3.1. Spectra Analysis

**Figure 1:**
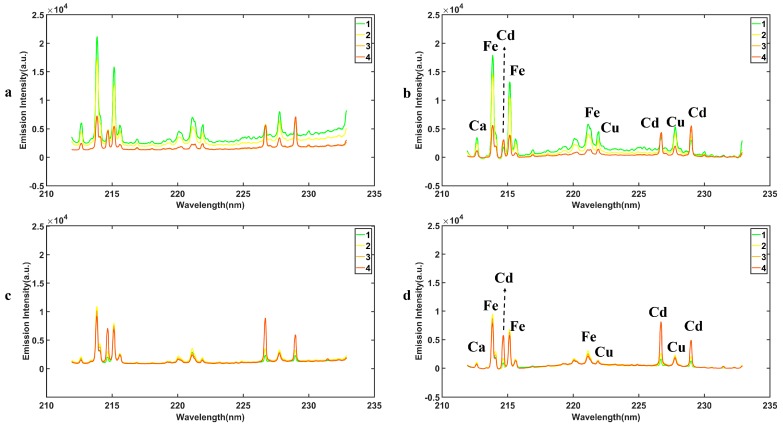
LIBS spectra. The average raw LIBS spectra of Cd concentration before pyrolysis (**a**) and after pyrolysis (**b**); LIBS spectra of samples containing Cd before pyrolysis (**c**) and after pyrolysis (**d**) preprocessed by adaptive iteratively reweighted penalized least squares (airPLS).

Figure 1 shows the average raw LIBS spectra of the four different Cd-stress group lettuces before and after pyrolysis (Figure 1a,b), and the spectra preprocessed by the airPLS method (Figure 1c,d). The LIBS spectra of different Cd concentration groups show similar tendencies which reveal that the samples had similar element and matrix compositions. Based on the Kurucz Database and National Institute of Standards and Technology (NIST) Atomic Spectra Database (ASD), three Cd emission lines (ionic emission lines Cd II 214.44 nm and Cd II 226.50 nm, atomic emission lines Cd I 228.80 nm) were observed in all Cd stress lettuce sample. As shown in Figure 1a,c, the baseline shifts occur in the LIBS spectra due to the random errors caused by the environment and instrument. Compared to Figure 1a, the baseline has been adjusted by the airPLS method in Figure 1c, which indicates that the data preprocessing is useful to decrease the random errors. As shown in Figure 1a,b, the pyrolysis of the sample makes huge contributions to correcting the baselines and enhancing the Cd peak signal in the spectral lines. That was because the pyrolysis process improved the properties of the sample and reduces the matrix effect.

**Figure 2:**
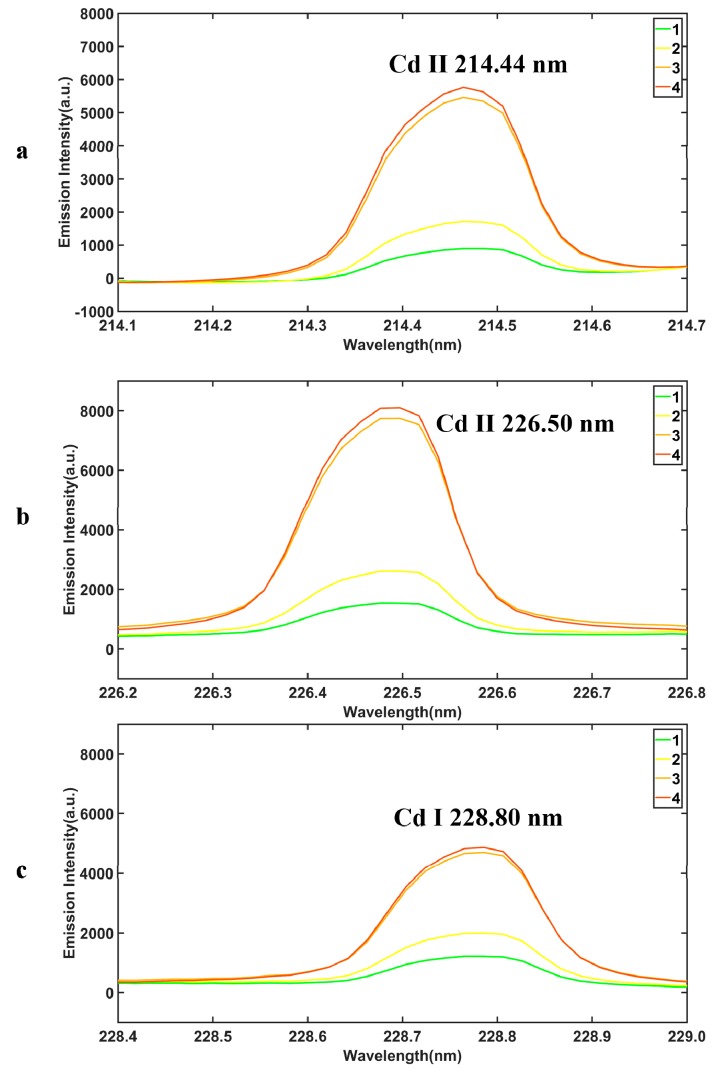
Three Cd spectral peaks of Figure 1d. Cd II 214.44 nm peak (**a**), Cd II 226.50 nm peak (**b**), Cd I 228.80 nm peak (**c**).

Figure 2 shows the details of three Cd spectral peaks from different groups in Figure 1d. After the pyrolysis of the sample and the preprocess of the airPLS method, the height of the Cd II 226.50 nm spectral peak is higher than the Cd II 214.44 nm peak and Cd I 228.80 nm. The Cd spectral peak of the different groups is related to the Cd content, the higher Cd content groups have higher Cd peaks.

### 3.2. Pyrolysis Evaluation

**Figure 3:**
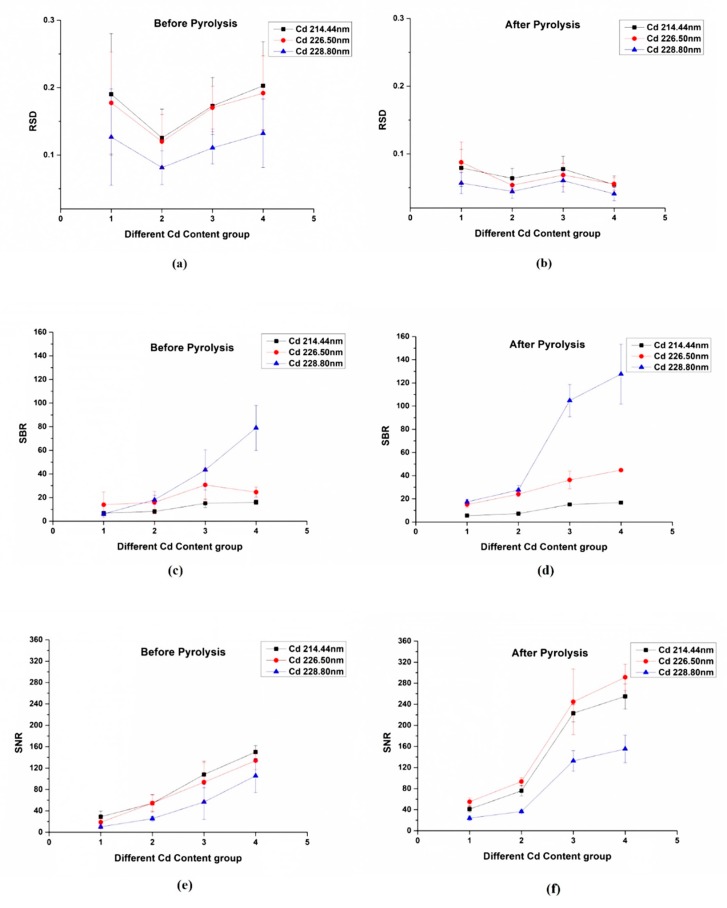
LIBS spectra. The relative standard deviation (RSD) of three Cd emission lines before pyrolysis (**a**) and after pyrolysis (**b**); the signal-to-background ratio (SBR) of three Cd emission lines before pyrolysis (**c**) and after pyrolysis (**d**); the signal-to-noise ratio (SNR) of three Cd emission lines before pyrolysis (**e**) and after pyrolysis (**f**). There are five samples in each group.

Figure 3 shows the three performance evaluation indexes including RSD, SBR and SNR of the four different Cd-stress group lettuces based on three Cd emission lines. The RSD of three Cd emission lines before and after pyrolysis are shown in Figure 3a,b, the SBR of three Cd emission lines before and after pyrolysis are shown in Figure 3c,d, the SNR of three Cd emission lines before and after pyrolysis are shown in Figure 3e,f. From Figure 3a,b, we found that the RSD of three Cd emission lines decreases in all four different Cd concentration groups after the process of pyrolysis, which indicates that the accuracy has been improved since the pyrolysis process is conducive to eliminate the matrix effect and reduce the random error. From Figure 3c,d, the value of SBR increased a lot on the Cd 228.80 nm emission line in the high Cd content group, while the SBR of Cd 214.44 nm and Cd 226.50 nm emission lines have no significant improvement, which indicates that the Cd 228.80 nm emission line makes a main contribution to the enhancement of the Cd signal in the LIBS spectra. From Figure 3e,f it is obvious that the SNR value of three emission lines have great improvement especially in the high Cd content group, which means the noise in the LIBS spectra have been eliminated a lot after the pyrolysis of the sample.

### 3.3. Multivariate Analysis

As an effective calibration method, the multivariate analysis is used to evaluate the Cd content of lettuce samples with multiple variables after the pretreatment of the LIBS spectra. Before the quantitative analysis, 20 samples were partitioned into a calibration set (13 samples) and a prediction set (7 samples). Selected variables and the full spectra were both applied to establish the models by using the partial least squares (PLS) regression and support vector regression (SVR) method.

#### 3.3.1. Modeling Based on Three Cd Emission Lines

The three Cd emission lines Cd II 214.44 nm, Cd II 226.50 nm and Cd I 228.80 nm were selected as the input variables to establish the multiple regression model. The results of the multiple regression model based on the three Cd emission line variables are shown in Table 2. As Table 2 shows, the model performs poorly with the *R_c_*^2^ value lower than 0.94 and *R_p_*^2^ value lower than 0.84 before the pyrolysis, which is mainly due to the loss of some background information and matrix information contained by other variables. After the preprocess of the raw data, the model improves a lot with the *R_c_*^2^ value of 0.9459 and *R_p_*^2^ value of 0.9154. However, the accuracy of the Cd content concentration still needs to be improved.

After the pyrolysis of the sample and acquired new LIBS data, the model preforms better than before with the *R_c_*^2^ value of 0.9907 and *R_p_*^2^ value of 0.9746 both in the calibration set and prediction set. Moreover, the preprocess of the data also achieves good performance especially in the prediction set. Therefore, the best model is based on the data after pyrolysis and preprocess, where the *R_c_*^2^ and *R_p_*^2^ are higher than 0.9915. After the pyrolysis treatment, the LODs were greatly reduced. This was because the pyrolysis treatment simplified the sample matrix composition, the background near the Cd signal decreased, and the SBR was higher. This indicates that the method of pyrolysis and preprocess methods are quite acceptable to the multiple regression model based on three Cd variables. The multivariate analysis based on the above model is conducive to develop the portable instrument and improve its performance for rapid detection of heavy metal Cd in the management of the lettuce market.

#### 3.3.2. Modeling Using Full Spectra

Compared to the model based on the three Cd emission lines, the model based on the full spectra obviously has a better performance on the Cd content concentration prediction because the full LIBS spectra contained all emission lines for elements and continuous background information. The range of the full spectra is from 211.92 nm to 232.90 nm with 1024 variables. The results of the multivariate analysis using the full spectra based on PLS and SVR models are shown in Table 3. As Table 3 shows, the PLS and SVR models based on the data acquired after the sample pyrolysis process achieve better performance than the models based on the un-pyrolysis data.

For the PLS model, the multivariate analysis based on the full spectra obtained poor performance with the *R_c_*^2^ value lower than 0.95 and *R_p_*^2^ value lower than 0.93 before the pyrolysis process. After applying the preprocessing algorithm, the PLS model performed better with the *R_p_*^2^ value higher than 0.96 while the *R_c_*^2^ value was lower than before. After the pyrolysis of the sample, the PLS model improves the performance with the *R_c_*^2^ value of 0.9973, RMSECV of 12.2 mg/kg in the calibration set, and *R_p_*^2^ value of 0.9973, RMSEP of 13.3 mg/kg in the prediction set. However, the preprocessing of data seems to achieve quite a little improvement on the PLS model after the pyrolysis.

For the SVR model based on the un-pyrolysis data with full spectra variables, the performance of the Cd content prediction is better than the PLS model with the *R_c_*^2^ value of 0.9887 and *R_p_*^2^ value of 0.9553. After the pyrolysis of the sample, the performance of the SVR model improved a lot especially the *R_p_*^2^ value of 0.9922 and RMSEP of 21.9 mg/kg in the prediction set, which indicates that the pyrolysis improves the property of the sample and reduces the matrix effects. However, the SVR model based on the preprocessing of the raw data achieves negative impact on the Cd content prediction, which seems that this preprocessing method may not be suitable for this model. The SVR model after pyrolysis and preprocessing seems overfitting in the calibration set, thus the *R_c_*^2^ value is as high as 1 and the *R_p_*^2^ value is quite low as 0.9693.

**Figure 4:**
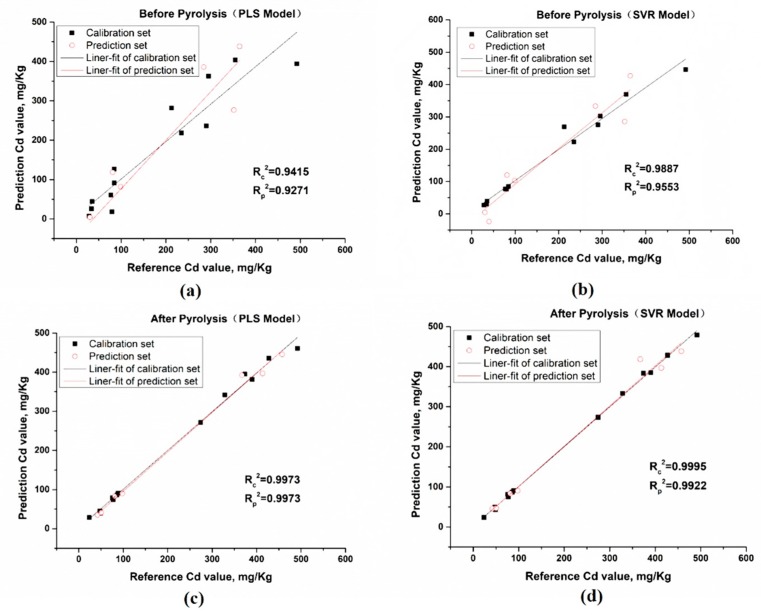
PLS model and SVR model. The PLS model before pyrolysis without preprocess (**a**) and SVR model before pyrolysis without preprocess (**b**); the PLS model after pyrolysis without preprocess (**c**) and SVR model after pyrolysis without preprocess (**d**).

Overall, the PLS and SVR model for the multivariable analysis based on the data after pyrolysis both achieve a good performance due to the elimination of the matrix effect. The full spectra provide a useful multi-variable which contains all the background and matrix information, and the method of pyrolysis make it possible to improve the properties of the sample and enhance the Cd signal in the LIBS spectra. However, the preprocessing algorithm of airPLS has no improvement for the above model. Therefore, the multivariable analysis based on the raw LIBS data after the pyrolysis is more suitable for accurate detection of the Cd content in lettuce leaves for laboratory research and food market regulation. Figure 4 shows the PLS and SVR model without the preprocessing method. 

According to the National Standards GB 2762-2017 in China, the maximum permitted concentration of Cd in vegetable leaves is 0.2mg/kg. For the multiple regression analysis based on the three Cd emission lines, LODs before pyrolysis is 12.1 mg/kg for raw spectra and 9.1 mg/kg for airPLS preprocessed spectra. LODs after pyrolysis is 1.1 mg/kg for raw spectra and 0.9 mg/kg for airPLS preprocessed spectra. For the multiple regression analysis based on full spectra, LOD of the PLS model after PLS is 0.8 mg/kg. Optimal results obtained by our previous lettuce Cd content based on the LIBS approach was LOD = 1.672 mg/kg [18]. The detection capability is inferior to the combination of LIBS and pyrolysis. Although the LODs of the combination of LIBS and pyrolysis did not meet the GB2762-2017, the pyrolysis greatly reduced the LODs, and the subsequent combination of other signal enhancement methods in further will be attempted to reduce the LODs to approach the GB requirement.

#### 3.3.3. Selecting Variables Using LASSO 

As shown above, Cd has limited emission lines and the majority of the full spectra are not sensitive to the Cd content. In addition, providing too much variables will easily lead to the model overfitting. Therefore, it is important to select a subset of the provided full spectra to create the model rather than using all of them. To this end, the penalty method and LASSO model are introduced.

LASSO will effectively choose a simpler model by forcing certain coefficients of some variables to be set to zero and remained variables are selected to construct the model. The alpha parameter of the LASSO model was determined by the cross validation of the calibration set. The selected lines and results of the LASSO model are shown in Table 4.

For the LASSO model based on the un-pyrolysis data, it selected seven variables with four of them close to the Cd emissions lines (Cd II 214.44 nm and Cd I 228.80), which verify the ability of LASSO to the selected key variables. Furthermore, the performance of the Cd content prediction is not as good as models using full spectra with the *R_c_*^2^ value of 0.9818 and *R_p_*^2^ value of 0.8828.

However, after the pyrolysis of the sample, the performance of the LASSO model improved significantly with the *R_p_*^2^ value of 0.9924 and RMSEP of 15.2 mg/kg in the prediction set. This improvement can be explained by the variable selection. For the dataset after pyrolysis, LASSO selected only four variables with three of them close to the Cd emissions lines, which indicates that the pyrolysis enhances the signal and eliminates the noise by reducing the matrix effects. In both datasets, the airPLS preprocessing has an insignificant impact on the results.

**Figure 5:**
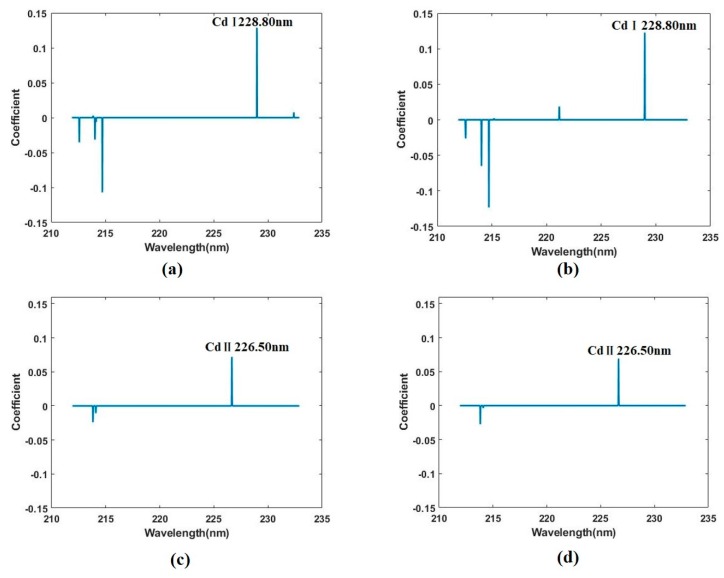
Least absolute shrinkage and selection operator (LASSO) coefficient. The coefficient of full spectra before pyrolysis without preprocess (**a**) and with preprocess (**b**); the coefficient of full spectra after pyrolysis without preprocess (**c**) and with preprocess (**d**).

The specific coefficients of selected variables are shown in Figure 5.

## 4. Conclusions

In this experiment, we improved the performance of the model by enhancing the heavy metal Cd signal in the laser-induced breakdown spectroscopy combined with pyrolysis process. Compared with chemometric methods with or without pyrolysis process, we achieved the rapid and accurate quantitative analysis for the Cd content in lettuce samples. A total of 20 sample solid pellets with Cd stress were prepared after the pyrolysis of lettuce powder. For the multivariable analysis, the multiple regression model based on the three Cd emission lines, the PLS and SVR model based on the full spectra all accomplish improvement compared to the data acquired from the un-pyrolysis sample. A total of three Cd emission lines Cd II 214.44 nm, Cd II 226.50 nm, and Cd I 228.80 nm were selected as three input variables to establish the multiple regression model, the one after the pyrolysis of the sample and preprocess of the raw data preformed best with the *R_c_*^2^ value of 0.9915 and *R_p_*^2^ value of 0.9969. For the PLS and SVR model based on the full spectra after the pyrolysis, the improvement created by the airPLS preprocessing method can be ignored. The PLS model without the preprocess achieved better performance with the *R_c_*^2^ and *R_p_*^2^ value of 0.9973, RMSECV of 12.2 mg/kg and RMSEP of 13.3 mg/kg, and the SVR model without the preprocess achieved better performance with the *R_c_*^2^ value of 0.9995, RMSECV of 5.5 mg/kg, *R_p_*^2^ value of 0.9922 and RMSEP of 21.9 mg/kg. Therefore, the PLS model based on the full spectra has better performance than the multiple regression model based on three Cd emission lines in both the calibration set and prediction set for Cd content prediction. For the LASSO model based on selected variables, the performance of the LASSO model improved significantly after the pyrolysis of the sample with the *R_p_*^2^ value of 0.9924 in the prediction set. Compared to the PLS and SVR model, the LASSO model achieved nearly the same accuracy but used less variables.

## Figures and Tables

**Table 1:** Cadmium (Cd) content of lettuce leaves before and after pyrolysis obtained by atomic absorption spectrophotometer (AAS) (mg/kg).

	Groups	1	2	3	4
before pyrolysis	Number	5	5	5	5
	Min	28.4	79.9	212.6	235.0
	Max	77.2	98.9	355.0	492.1
	Mean	40.8	85.8	287.6	360.8
	S.D.	16.7	6.8	45.3	91.0
after pyrolysis	Min	23.8	77.2	274.8	329.0
	Max	75.8	97.9	428.2	493.0
	Mean	48.2	86.3	367.4	423.6
	S.D.	15.3	7.0	50.8	61.4

Note: Group 1 represents 10 µM Cd stress; Group 2 represents 30 µM Cd stress; Group 3 represents 60 µM Cd stress; Group 4 represents 100 µM Cd stress. These expressions apply to the full text.

**Table 2:** The results for multiple regression analysis based on three Cd emission lines.

Sample	Variables (nm)	Calibration Set (*R_c_*^2^)		Prediction Set (*R_p_*^2^)		LOD (mg/kg)	
		Raw	airPLS	Raw	airPLS	Raw	airPLS
Before Pyrolysis	214.44, 226.50, 228.80	0.9387	0.9459	0.8399	0.9154	12.1	9.1
After Pyrolysis	214.44, 226.50, 228.80	0.9907	0.9915	0.9746	0.9969	1.1	0.9

**Table 3:** The results of multivariate analysis based on full spectra by partial least squares (PLS) and support vector regression (SVR) models.

Sample Type	Preprocess	Model	Parameter	Calibration Set		Prediction Set		LOD (mg/kg)
				*R_c_* ^2^	RMSECV	*R_p_* ^2^	RMSEP	
Before Pyrolysis	/	PLS	n = 5	0.9415	48.9	0.9271	75.9	8.9
	/	SVR	*gam* = 8.6 × 10^4^ *sig2* = 1.9 × 10^5^	0.9887	21.4	0.9553	49.2	/
	airPLS	PLS	n = 5	0.9372	51.1	0.9616	44	8.4
	airPLS	SVR	*gam* = 1.7 × 10^2^ *sig2* = 7.9 × 10^3^	0.9854	25.0	0.9546	42.9	/
After Pyrolysis	/	PLS	n = 2	0.9973	12.2	0.9973	13.3	0.8
	/	SVR	*gam* = 8.4 × 10^5^ *sig2* = 6.2 × 10^6^	0.9995	5.5	0.9922	21.9	/
	airPLS	PLS	n = 2	0.9976	11.6	0.9966	14.9	0.9
	airPLS	SVR	*gam* = 7.3 ×10^8^ *sig2* = 4.1 × 10^7^	1	0.0	0.9693	46.3	/

Parameter **n** in the PLS model means principal component; parameter ***gam*** and ***sig2*** in the SVR model means the optimal parameter for cross-validation.

**Table 4:** The results for LASSO model based on selected variables.

Sample Type	Preprocess	Variables	Calibration Set		Prediction Set	
			*R_c_* ^2^	RMSECV	*R_p_* ^2^	RMSEP
Before Pyrolysis	/	212.40, 213.68, 213.85, 213.95, 214.53, 228.79, 232.19;	0.9818	19.2	0.8828	47.2
	airPLS	212.38, 212.40, 213.85, 214.53, 215.00, 220.97, 228.77, 228.79;	0.9835	18.2	0.9050	42.4
After Pyrolysis	/	213.62, 213.89, 226.46, 226.48;	0.9977	7.8	0.9924	15.2
	airPLS	213.62, 213.89, 226.46, 226.48;	0.9976	7.9	0.9926	15.0
